# Visual Tracking Based on an Improved Online Multiple Instance Learning Algorithm

**DOI:** 10.1155/2016/3472184

**Published:** 2015-12-30

**Authors:** Li Jia Wang, Hua Zhang

**Affiliations:** ^1^Department of Information Engineering and Automation, Hebei College of Industry and Technology, Shijiazhuang 050091, China; ^2^Faculty of Electrical & Electronics Engineering, Shijiazhuang Vocational Technology Institute, Shijiazhuang 050081, China

## Abstract

An improved online multiple instance learning (IMIL) for a visual tracking algorithm is proposed. In the IMIL algorithm, the importance of each instance contributing to a bag probability is with respect to their probabilities. A selection strategy based on an inner product is presented to choose weak classifier from a classifier pool, which avoids computing instance probabilities and bag probability *M* times. Furthermore, a feedback strategy is presented to update weak classifiers. In the feedback update strategy, different weights are assigned to the tracking result and template according to the maximum classifier score. Finally, the presented algorithm is compared with other state-of-the-art algorithms. The experimental results demonstrate that the proposed tracking algorithm runs in real-time and is robust to occlusion and appearance changes.

## 1. Introduction

The purpose of visual tracking is to estimate the state of a target from frame to frame. It has been widely studied in computer vision, for example, surveillance, robot navigation, and human-computer interaction [1]. Although various tracking algorithms have been proposed [2–11], visual tracking is still a very challenging task due to appearance changes caused by pose, illumination, occlusion, and motion [1].

An effective appearance model is important in the tracking process. The appearance model represents a target by using information extracted from the target region. Since color feature is readily accessible from an image, it is widely used to model a target's appearance [3]. One of the best methods for color-based visual tracking is to realize the MS algorithm [4, 5]. The color histogram of a target is learned in the first frame for color based object tracking algorithm, and then the target is searched in the subsequent frames by measuring the similarity between two histograms. The MS algorithm has been successfully used in visual tracking due to its similarity, efficiency, and robustness [4, 5]. However, the color histogram can only describe the color distribution in a region but ignore the spatial distribution. Therefore, its representation ability will significantly decline when there are partial occlusions or similar objects. As a result, a color based tracker may wrongly detect the “bad” candidate as tracking result. To overcome the drawback, parts-based color histograms were adopted [6]. The method is robust to occlusions as it combines the color histograms of multiple parts. Recently, compressed sensing theory has attracted much attention in computer vision, for example, face recognition [7], denoising and in-painting [8], and visual tracking [9–12]. It is demonstrated that a compressed sensing based tracking method can cope with partial occlusions, illumination changes, and pose variations [11].

The generative appearance models mentioned above are learned in the first frame. Therefore, they cannot deal with major appearance changes as the track evolves. Discriminative algorithms, which can change appearance model evolution, were proposed [13]. The main idea of these appearance models is to consider the tracking problem as a binary classification task by continuously updating a classifier [14]. The best way to implement this task is to design appearance models for the target and background [14] and after that learn a discriminative classifier according to the models. Grabner [15] proposed an online boosting algorithm to learn a discriminative appearance model for visual tracking. The tracker took the current tracking location as one positive example and extracted negative samples from neighborhood around the tracking location. However, the results often drifted away as the appearance model was updated with a suboptimal positive example. Zhang et al. [16] implemented a real-time compressed tracking algorithm. Multiple positive examples and negative ones were cropped around the current tracking location to update a discriminative classifier. However, the ambiguity problem may occur and confuse the classifier [17]. To deal with this problem, Grabner et al. [17] proposed a semisupervised boosting approach where only the samples from the first frame were labeled and the subsequent training examples were left unlabeled. The method achieved good performance even if the target disappears from the field of view of a camera. However, the tracker failed when there was great interframe motion.

The methods of tracking-by-detection have progressed in the past few decades. These methods train a classifier by adopting positive samples and negative ones extracted from the current frame. In the tracking process, some candidates around the old object location are extracted, and the trained classifier is employed to find the sample with the maximum classifier score. In fact, Viola et al. [18] argued that the inherent ambiguities included in object detection would cause difficulty for traditional learning methods. To handle the problem, the multiple instance learning (MIL) method was proposed for object detection [18, 19] and object recognition [20–22]. Studies show that the technique can cope with partial occlusions and ambiguities [22, 23]. The basic idea of the MIL method is that examples are called bags and some instances are included in these bags [14]. The bags instead of the individual instance are labeled. A bag is positive if at least one instance is positive. In contrast, a bag is negative when all of the instances are negative. Normally, the positive bag is constructed by the instances sampled around the labeled object. The MIL based tracking method can cope with the ambiguity problem by finding out the most “correct” instance in each positive bag [19]. However, the MIL trackers still have some shortcomings, for example, a heavy computational load, tracking drift, and failure to handle appearance variations. To address these problems, Zhang and Song [24] proposed an improved MIL tracker by weighting instance probabilities. Zhang and Song used an efficient online approach to maximize the bag likelihood function, which resulted in robust and fast tracking. However, when a bag probability is computed, the included instances are assigned different weights based on their distance to the previous tracking location. As a result, the target will be lost when drift occurs, because the real instance far away from the previous tracking location is assigned a large weight. Xu et al. [25] presented an efficient MIL tracker by using a Fisher information criterion, which yielded robust performance. Zhou et al. [26] implemented online visual tracking by using MIL with instance significance estimation. The method provided each instance a significance-coefficient which represents its contribution to the bag likelihood. The method dealt with the drift problem to some extent. In general, these MIL trackers successfully deal with slight appearance variations and achieve a robust and fast tracking. However, these trackers fail to handle strong ambiguity, for example, large appearance variations, and tracking drift.

In this study, to deal with the above problems, we propose an improved online multiple instance learning algorithm (IMIL) for object tracking. The IMIL algorithm has advantages over the other MIL trackers in terms of efficiency, accuracy, and robustness. First, to reduce the computational cost, an inner product based selection method is employed to choose the weak classifiers from a classifier pool. The selection strategy avoids computing the instance probabilities and bag probability *M* times. Consequently, compared with the MIL, the IMIL runs in real time. Second, to generate a bag probability, the importance of each instance is with respect to their probabilities. Compared with the WMIL tracker, the strategy avoids introducing interference from the background and then deals with the drift problem. Third, to cope with the variations on illumination and pose, a feedback strategy is proposed to update the parameters of classifiers. In the strategy, the learning rate is tuned by considering the relationship between the maximum similarity score and two given thresholds after detecting the object. Therefore, using the proposed update strategy, the IMIL handle the variations on appearance and illumination.

The rest of this paper is organized as follows.  Section 2 details the proposed tracking algorithm; Section 3 presents the experimental results of the proposed tracker compared with other trackers; the paper concludes with a short summary in Section 4.

## 2. The IMIL Based Tracking Algorithm

**Figure 1:**
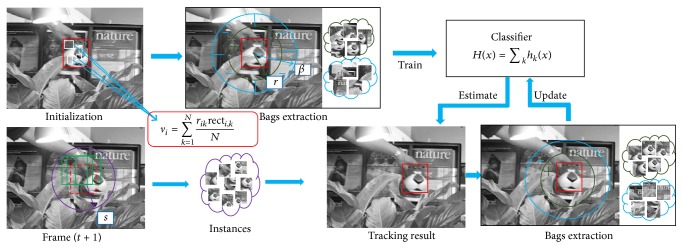
The flow chart of object tracking by using IMIL algorithm. The locations of the solid red rectangles are the tracking results. The areas of the brown circles are the ones for selecting instances to generate the positive bags. The areas of the blue circles are the ones for the negative bags. The location of the dashed rectangle in the *t* + 1 frame is the tracking result from the *t* frame. The green rectangles in the *t* + 1 frame are the candidate samples extracted from a small neighborhood of the result in the *t* frame.

An improved online multiple instance learning algorithm is proposed for object tracking in video sequences. The basic flow of the IMIL algorithm is shown in Figure 1. Each instance is represented by a set of compressed Haar-like features [14, 24]. Let *l*_*t*_(*x*) ∈ *R* ^2^ denote the location of each instance *x* in the *t*th frame. It is assumed that the tracker can detect the object at the location *l*_*t*_^*∗*^. Once the object is detected, a set of instances *X* ^*r*^ = {*x* : ‖*l*(*x*) − *l*_*t*_^*∗*^‖ < *r*} are cropped to construct a positive bag. In contrast, the instances for negative bag are cropped from an annular region *X* ^*r*,*β*^ = {*x* : *r* < ‖*l*(*x*) − *l*_*t*_^*∗*^‖ < *β*}, *r* < *β*. The extracted positive bag and negative one are adopted to train weak classifiers. Then, a strong classifier is generated by selecting the weak classifiers with stronger classifying ability. For the *t* + 1 frame, we assume that the object appears within a radius *s* of the tracking location in the *t* frame. Thus, some candidate samples *X* ^*s*^ = {*x* : ‖*l*_*t*+1_(*x*) − *l*_*t*_^*∗*^‖ < *s*}, *r* < *s* < *β*, are cropped within a search radius *s* centering at the previous location *l*_*t*_^*∗*^. The learned strong classifier estimates *p*(*y* = 1∣*x*) for all samples *x* ∈ *X* ^*s*^ and updates the tracking location according to *l*_*t*_^*∗*^ = *l*(arg⁡max_*x*∈*X*^*s*^_⁡*p*(*y* = 1∣*x*)). Furthermore, the classifier updates the appearance model after tracking a new location.

### 2.1. Online MIL Boosting Algorithm

Babenko et al. [14] proposed an online MIL Boosting method for visual tracking. The details of the MIL Boosting tracker are shown in Algorithm 1. The training data is {(*X*_1_, *y*_1_),…, (*X*_*n*_, *y*_*n*_)}, where *X*_*i*_ = {*x*_*i*1_,…, *x*_*im*_} is a bag constructed by instances *x*_*i*_ and *y*_*i*_ is the bag label according to *X*_*i*_. The key of the MIL Boosting method is to design a strong classifier *H*(·) = ∑_*k*=1_^*K*^ *h*_*k*_(·). *h*_*k*_(·) is a weak classifier relating to a sampled instance (represented by Haar-like feature *V*_*k*_) in the positive bag. The weak classifier *h*_*k*_(·) is assumed to be Gaussian distributed with four parameters (*μ*_*k*_^1^, *σ*_*k*_^1^, *μ*_*k*_^0^, *σ*_*k*_^0^), where *p*(*v*_*k*_(*x*)∣*y* = 1) ~ *N*(*μ*_*k*_^1^, *σ*_*k*_^1^) and *p*(*v*_*k*_(*x*)∣*y* = 0) ~ *N*(*μ*_*k*_^0^, *σ*_*k*_^0^). The weak classifier returns the log odds ratio:

(1) $$\begin{array}{c} h_{k}\left(\;x\;\right)=\log\left(\;\frac{p\left(y=1\;|\;v_{k}\left(x\right)\right)}{p\left(y=0\;|\;v_{k}\left(x\right)\right)}\;\right). \end{array}$$

When the strong classifier receives new data {(*X*_1_, *y*_1_),…, (*X*_*n*_, *y*_*n*_)}, the instance probability is modeled as

(2) $$\begin{array}{c} p\left(\;y_{i}\;|\;x_{ij}\;\right)=\sigma \left(\;H\left(\;x_{ij}\;\right)\;\right), \end{array}$$

where *σ*(*x*) = 1/(1 + exp⁡(−*x*)) is the sigmoid function. Then, the *i*th bag probability is modeled by adopting the Noisy-OR model [18]:

(3) $$\begin{array}{c} p\left(\;y_{i}\;|\;X_{i}\;\right)=1-\prod_{j}\left(\;1-p\left(\;y_{i}\;|\;x_{ij}\;\right)\;\right). \end{array}$$

The MIL tracker learns a pool of *M* candidate weak classifiers. Then, the *K* (<*M*) weak classifiers are selected from the candidate pool by maximizing the log likelihood of bags:

(4) $$\begin{array}{c} h_{k}=\underset{h\in \left\{h_{1},\ldots ,h_{M}\right\}}{\mathrm{argmax}}L\left(\;H_{k-1}+h\;\right), \end{array}$$

where *L* = ∑_*i*_(*y*_*i*_log⁡(*p*_*i*_) + (1 − *y*_*i*_)log⁡(1 − *p*_*i*_)) is the bag log-likelihood function and *H*_*k*−1_ = ∑_*k*=0_^*k*−1^ *h*_*k*_. Finally, the selected *K* weak classifiers generate a strong classifier *H* to determine the new tracking location with the maximum score.

After detecting the object in the new frame, the parameters of the weak classifiers are updated online:

(5) μi1⟵λui1+1−λμ1σi1⟵λσi12+1−λσ12+λ1−λμi1−μ12,

where *λ* is a learning rate. It specifies the importance of the tracking result and the template.

The MIL Boosting algorithm deals with the suboptimal problem included in the weak classifiers. However, the bag probability computed by using the Noisy-OR model makes the MIL tracker easily select less effective features and confuse the classifier [27]. Another disadvantage of the MIL tracker is that all of the instance probabilities and the bag probabilities must be updated *M* times after selecting a feature, which results in a heavy computational load [24]. Moreover, the weak classifiers update their parameters with fixed learning rate, which is sensitive to the occlusion and variations on illumination and appearance.

### 2.2. Bag Probability

To overcome the drawback mentioned above, the IMIL tracker is proposed. It is assumed that the bag's probability depends on the instance probability equally [28]. The importance of each instance contributing to a bag probability mainly depends on the instance probability. The bigger the instance probability is, the more it contributes to the bag probability. We use an equally weighted sum of instance probabilities to yield superior performance. The positive bag probability is defined as follows:

(6) $$\begin{array}{c} p\left(\;y=1\;|\;X_{i}\;\right)=\overset{N-1}{\underset{j=0}{\sum }}p\left(\;y=1\;|\;x_{ij}\;\right). \end{array}$$

**Figure 2:**
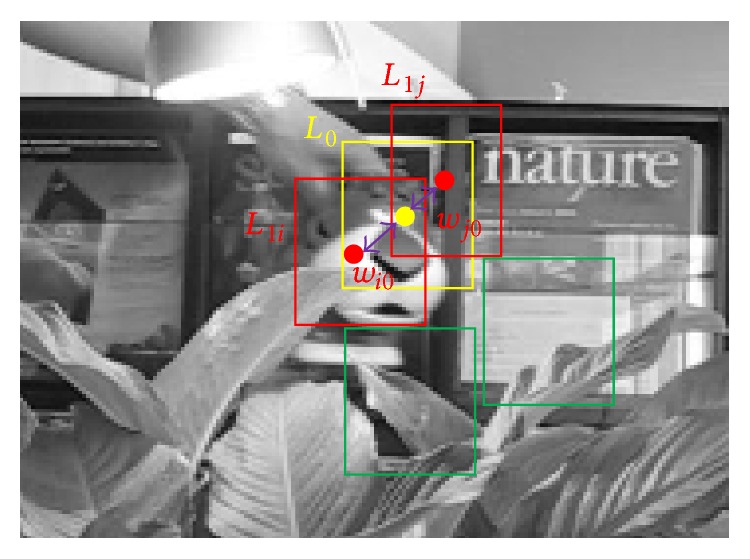
The illustration of constructing the bag probability. The location of the yellow rectangle is for the current tracking results. The red rectangles are for the positive instances, while the green rectangles are for the negative ones.

Compared with the Noisy-OR model and the WMIL tracker, our method computes the bag probability according to each instance probability. In the WMIL tracker, the positive instances are assigned weights to generate the bag probability according to the Euclidean distances between the locations of each instance and the current tracking location *p*(*y* = 1∣*X*_*i*_) = ∑_*j*=0_^*N*−1^ *w*_*j*0_ *p*(*y* = 1∣*x*_*ij*_) [24]. The nearer the distance is, the more important the instance probability is. However, if the tracking location *L*_0_ drifts away from the real target position (see Figure 2), the instance *L*_1*i*_ far from the current tracking location will be assigned a smaller weight than that for the near instance *L*_1*j*_ due to their distances. As a result, the instance *L*_1*j*_ contributes more to the bag probability than the instance *L*_1*i*_, which is contrary to the fact. As a result, the obtained bag probability will contain information from the background and finally lead to a tracking failure. In our method, the instance's importance is determined by their probabilities. The instance whose probability is bigger will contribute more to the bag probability. Using the strategy, the tracker achieves superior performance.

### 2.3. Selection Strategy

The MIL tracker often suffers from a heavy computational load as it selects the weak classifier *h*_*k*_ with stronger classifying ability from a weak classifier pool *ϕ* = {*h*_1_,…, *h*_*M*_} by maximizing the log-likelihood function. To realize real time visual tracking, a more efficient criterion based on the inner product is employed to select weak classifiers from the classifier pool [24]. Using this criterion, the instance probabilities and bag probabilities do not need to be computed *M* times after selecting one weak classifier. Inspired by this, we select the weak classifier *h*_*k*_ as follows:

(7) $$\begin{array}{c} h_{k}=\underset{h\in \phi }{\mathrm{argmax}}{\left.\;\langle \;h,\nabla L\left(\;H\;\right)\;\rangle \;\right|}_{H=H_{k-1}}, \end{array}$$

where 〈*h*, ∇*L*(*H*)〉 = 1/(*N* + *L*)∑_*j*=0_^*N*+*L*−1^ *h*(*x*_*ij*_)∇*L*(*H*)(*x*_*ij*_),

(8) ∇LHxij=yiσHxij1−σHxij∑m=0N−1σHxim−1−yiσHxij1−σHxij∑m=NN+L−11−σHxim,

where *p*(*y*∣*x*_*ij*_) = *σ*(*H*(*x*_*ij*_)). The IMIL method is shown in Algorithm 2.

### 2.4. Weak Classifiers

The weak classifiers of the MIL update their four parameters with fixed weights [16], which may introduce errors when there are inaccurate tracking results. Normally, the tracking process is formulated as a searching problem which aims to select the candidate with the maximum classifier score (*H*_max_). Usually, the most “correct” samples detected by the tracker are similar in the most frames. However, when there is an occlusion or a great interframe motion, the update strategy will introduce errors from background and lead to a suboptimal result in the rest of the frames. Consequently, drift will occur or the target will be lost.

To avoid the problems mentioned above, a feedback strategy is proposed for updating the classifiers. Our goal is to detect the candidate area not only the most similar to the foreground but also the most dissimilar to the background. The maximum classifier score *H*_max_ obtained by applying the strong classifier denotes the similarity between the tracking result and target. In our method, TH_*l*_ and TH_*s*_ are set as higher threshold and lower threshold, respectively. To make sure the classifier is far from the background, the update strategy changes the learning rate *λ* considering the relationship between the *H*_max_ and TH_*l*_ or TH_*s*_:

(9) $$\begin{array}{c} \lambda =\left\{\begin{array}{cc} 0.85, & H_{max}>{\text{TH}}_{l}\\ 0.25, & {\text{TH}}_{s}<H_{max}<{\text{TH}}_{l}\\ 0.05, & H_{max}<{\text{TH}}_{s}, \end{array}\right. \end{array}$$

where *λ* is the learning rate. In this strategy, it specifies the importance of the tracking result, while 1 − *λ* specifies the importance of the template. *H*_max_ > TH_*l*_ means that the candidate area is more similar to the template (foreground), while *H*_max_ < TH_*s*_ means that the candidate area is more similar to the background. Considering the relationships between the *H*_max_ and the two thresholds, two weights are assigned to the tracking result and template to specify their importance when the classifier is updating.

The feedback strategy can deal with appearance changes and avoid excessive updates as the track evolves. When the target is successfully tracked, the maximum classifier score *H*_max_ is greater than TH_*l*_, which means that the similarity between the tracking result and template is very high. Then, the weight of the tracking result is increased to the largest value to cope with appearance variations. In other words, the new classifier depends mainly on the tracking result. When there is a serious occlusion or the target is lost, the most “correct” target includes numerous background information. The maximum classifier score *H*_max_ is less than the lower threshold TH_*s*_, which means that the similarity between the tracking result and template is very low. Then, the weight of the tracking result should be a small value to avoid introducing more errors to the classifier. As a result, the new classifier depends mainly on the template. When there are partial occlusions or some appearance variations, the *H*_max_ is between the lower threshold and the higher one. In such a case, the classifier should update according to the tracking result and the template simultaneously.

## 3. Experiments

**Figure 3:**
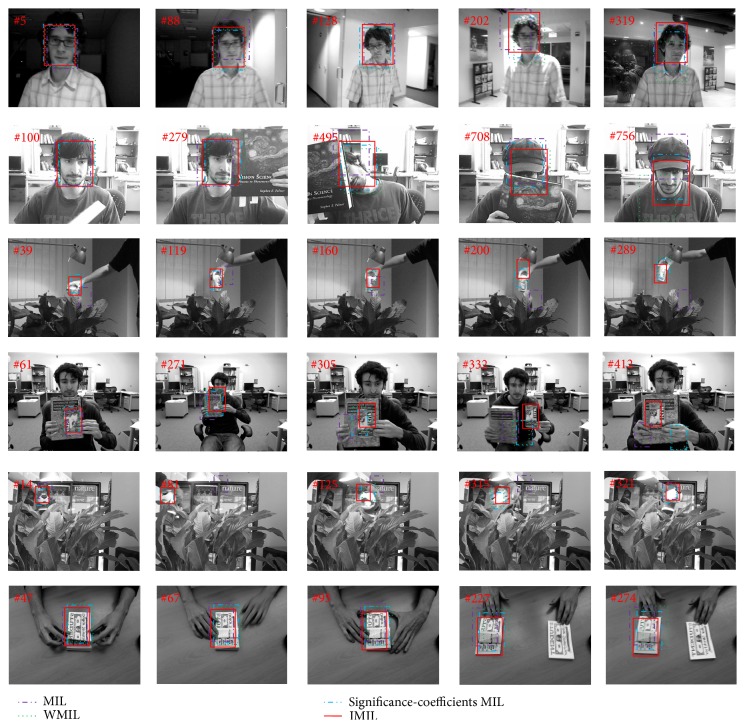
Tracking object location by using MIL [14], WMI [24], significance-coefficients MIL [26], and IMIL. The video sequences are “David indoor,” “Occluded face,” “Coke can,” “Cliff bar,” “Tiger 2,” and “Coupon book” from the top to the bottom.

We compared the proposed object tracking algorithm (IMIL) with the 3 latest trackers on 6 challenging video sequences. The three trackers are online MIL Boosting tracker (MIL) [14], WMIL tracker [24], and significance-coefficients MIL [26]. For the compared trackers, the binary code released by the authors is used [26]. The six video sequences are “David indoor” [24], “Occluded face” [24], “Tiger 2” [24], “Cliff bar” [24],“Coke can” [14], and “Coupon book” [26] (Figure 3). Using these video sequences, we evaluate the IMIL tracker's ability of handling the problems of illumination changes, occlusion, pose variations, and appearance changes. There are serious illumination variations in “David indoor” and “Coke can.” And the “can” moves fast in the “Coke can” video. In the sequences “Occluded face 2” and “Tiger 2,” the face is often occluded by a book and the “Tiger” is often occluded by the leaves. Moreover, there are still pose changes in these video sequences. The pose variations exist in the video “Cliff bar,” while the appearance of the “dollar” changes in the video “Coupon book.” The proposed IMIL is implemented in the MATLAB and run on a core 2 CPU, 2.33 GHz, and 2 GB RAM computer.

### 3.1. Parameters Setting

For the online MIL Boosting tracker [14], the search radius *s* is set to 35, and about 1000 samples are extracted for detecting the object location. Set *r* = 4 and sample about 45 positive instances for generating the positive bag; *β* = 50; the negative instances in the negative bag are about 65. The number of the candidate weak classifiers is 250. 50 classifiers are selected from weak classifier pool to generate a strong classifier. The learning rate of the weak classifier is 0.85. For the WMIL tracker [24], set *s* = 25 to search the target; *r* = 4 to crop the positive instances; *a* = 2*r* and *β* = 1.5*s* to determine the negative instances. The number of candidate weak classifiers is set to be *M* = 150. 15 weak classifiers are selected from classifiers pool to generate a strong WMIL classifier. The learning parameter is still 0.85. For the significance-coefficients MIL [26], set *r* = 4 and *β* = 50 to generate the positive and negative bags; the learning rate is set to be 0.85. The number of the candidate weak classifiers is *M* = 150, and 15 weak classifiers are selected for generating a strong classifier. For the IMIL tracker, set *s* = 25 for detecting the target; *r* = 4 is set for sampling positive instances; *a* = 2*r* and *β* = 1.5*s* are set for extracting negative instances. To generate a strong classifier, 30 weak classifiers are selected from the classifier pool which includes 120 weak classifiers.

### 3.2. Tracking Object Location

We perform our experiments on the above 6 video sequences. For all sequences, the images are converted into gray scale before processing. For each sequence, the classifier is learned in the first frame. Then, the locations in the subsequent frames are tracked by these trackers. For updating the classifier, the proposed IMIL algorithm tunes the parameters of the tracking result and template according to the maximum similarity in the tracking process. Therefore, compared with MIL, WMIL, and significance-coefficients MIL trackers, the IMIL tracker achieves the best performance when there are appearance changes, pose and illumination variations. Furthermore, the IMIL method computes a bag probability according to instance probability. Therefore, an instance contributes more to a bag probability if the instance probability is bigger. As a result, the IMIL tracker overcomes the tracking drift problem existing in the WMIL when there is fast moving (e.g., Coke can) in the tracking process.

### 3.3. Quantitative Analysis

We employ the center location error and overlap rate to evaluate the performance of our method. The center location error measures the position error between central locations of the tracking results and the centers of the ground truth. The overlap rate measures the tracking result on the area of overlap with ground truth bounding boxes [29]. The overlap rate is defined as score = area(*R*_*T*_∩*R*_*G*_)/area(*R*_*T*_ ∪ *R*_*G*_). The *R*_*T*_ is the area of the tracking result, while the *R*_*G*_ is for the ground truth bounding boxes. The tracking result with the overlap rate exceeding 50% is considered to be a correct detection.

**Figure 4:**
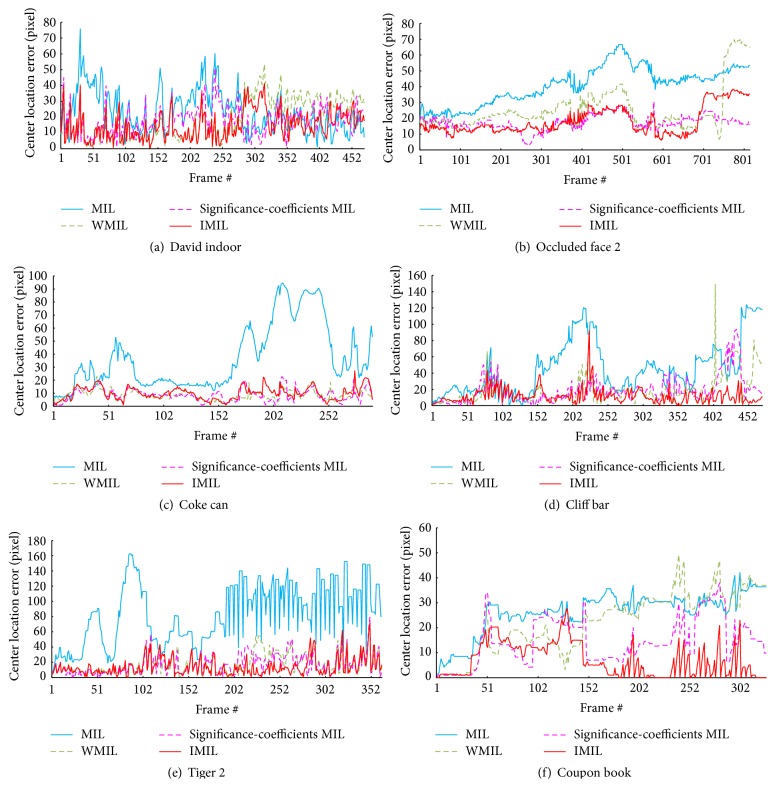
The center location errors for the six videos.

The center location errors for all the trackers are shown in Figure 4. The average center location errors are detailed in Table 1. The smaller the average center location error is, the better the tracking algorithm performs. Bold indicates the best performance. The overlap rate for all the trackers are detailed in Table 2. Bold indicates the best performance. In the IMIL tracker, the bag probability is calculated according to instance probability. When there is tracking drift or fast moving, the bag probability mainly depends on the instance whose probability is the largest. Therefore, the drift problem can be corrected. Moreover, the learning rate is tuned according to the tracking result. As a result, the IMIL handles the problem of occlusion and variations on illumination and pose. Therefore, compared with the other MIL based trackers, the IMIL tracker is robust to the problem of drift, occlusion, illumination variations, and pose changes. The experimental results show that IMIL algorithm performs well.

### 3.4. Computational Cost

In this section, we compared our method with MIL, WMIL, and significance-coefficients MIL in terms of computational cost. The average computing time processing an image measures the computational cost of these algorithms. The average computing time is defined as *T* = *T*_all_/*N*_frames_. *T*_all_ is the total computing time processing the whole video sequence. *N*_frames_ is the number of frames in the sequences. *T* is the obtained average computing time. The computational cost of these methods is affected by three main factors: the strategy for selecting weak classifiers, the number of weak classifiers, and the number of the selected weak classifiers for generating a strong classifier. The IMIL tracker is in the lowest computational load due to its advantages as follows: (1) the proposed criterion for selecting weak classifiers avoids computing the instance probabilities and bag probability *M* times before choosing one weak classifier; (2) about 120 weak classifiers are learned and 30 weak classifiers are chosen for generating a strong classifier (for MIL and WMIL, the number of the learned weak classifiers is 150 and that of the selected classifiers is 50). The average computational cost for different algorithms conducted on the 6 video clips is shown in Table 3. The results show that the IMIL is improved with lower computational time.

## 4. Conclusion

In this paper, we presented an improved online multiple instance learning algorithm for visual tracking. A feedback scheme was used to update the parameters of the classifiers, which can handle the appearance changes caused by pose, illumination, and occlusion. We equally summed the instance probabilities to generate a bag probability. The method can avoid introducing the information from background and yield superior performance. A more efficient criterion was proposed to select weak classifiers, which avoided computing the instance probabilities and the bag probabilities *M* times after selecting one weak classifier. Finally, numerous experiments on challenging video sequences demonstrated that the proposed algorithm performs well in terms of efficiency, accuracy, and robustness.

## Figures and Tables

**Algorithm 1:**
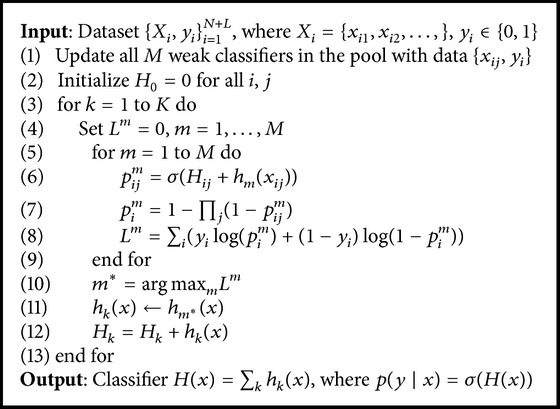
Online MIL Boosting.

**Algorithm 2:**
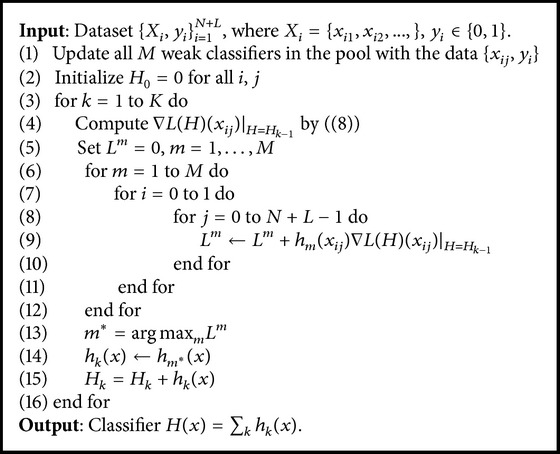
Online IMIL.

**Table 1:** The average center location errors for all of the trackers. Bold indicates the best performance.

Video clip	MIL	WMIL	Significance-coefficients MIL	IMIL
David indoor	23.631	19.435	17.716	**14.739**
Occluded face 2	33.805	22.165	15.747	**15.368**
Coke can	37.158	**8.720**	8.777	9.905
Cliff bar	43.994	18.225	18.882	**11.97**
Tiger 2	88.851	17.844	17.279	**13.972**
Coupon book	26.960	22.232	14.774	**7.179**

**Table 2:** The overlap rate for all of the trackers. Bold indicates the best performance.

Video clip	MIL	WMIL	Significance-coefficients MIL	IMIL
David indoor	0.65	0.84	0.90	**0.92**
Occluded face 2	0.78	0.71	0.78	**0.85**
Coke can	0.45	0.72	0.68	**0.86**
Cliff bar	0.53	0.64	0.75	**0.82**
Tiger 2	0.36	0.59	0.67	**0.79**
Coupon book	0.75	0.86	**0.88**	0.87

**Table 3:** The average computational cost (s) for different algorithms conducted on 6 video clips.

Video clip	MIL	WMIL	Significance-coefficients MIL	IMIL
David indoor	1.122	0.109	1.021	0.084
Occluded face 2	1.601	0.104	1.108	0.087
Coke can	1.089	0.104	0.81	0.104
Cliff bar	1.092	0.098	0.962	0.079
Tiger 2	1.092	0.103	1.096	0.094
Coupon book	1.164	0.103	0.978	0.086
